# Syntax and the brain: language evolution as the missing link(ing theory)?

**DOI:** 10.3389/fpsyg.2024.1445192

**Published:** 2024-10-25

**Authors:** Antonio Benítez-Burraco, Ljiljana Progovac

**Affiliations:** ^1^Department of Spanish, Linguistics, and Theory of Literature (Linguistics), Faculty of Philology, University of Seville, Seville, Spain; ^2^Department of English, College of Liberal Arts and Sciences, Wayne State University, Detroit, MI, United States

**Keywords:** syntax, neurolinguistics, language evolution, aggression, language disorders, cortico-striatal networks

## Abstract

**Introduction:**

This paper provides proof of concept that neurolinguistic research on human language syntax would benefit greatly by expanding its scope to include evolutionary considerations, as well as non-propositional functions of language, including naming/nicknaming and verbal aggression. In particular, an evolutionary approach can help circumvent the so-called granularity problem in studying the processing of syntax in the brain, that is, the apparent mismatch between the abstract postulates of syntax (e.g. Tense Phrase (TP), Determiner Phrase (DP), etc.) and the concrete units of neurobiology (neurons, axons, etc.).

**Methods:**

First, we decompose syntax into its evolutionary primitives, identifying one of the earliest stages as a simple, flat combination of just one verb and one noun. Next, we identify proxies (“living fossils”) of such a stage in present-day languages, including compounds and small clauses, lacking at least some layers of structure, e.g. TPs and DPs. These proxies of ancestral language have been subjected to fMRI neuroimaging experiments.

**Results:**

We discuss the finding that less hierarchical small clauses, in contrast to full sentences with TPs and DPs, show reduced activation in the left Broca’s area (BA) 44 and the right basal ganglia, consistent with the hypothesis that more recent, more elaborate syntax requires more connectivity in the Broca’s-basal ganglia network, whose neuronal density has been significantly enhanced in recent evolution, implicating mutations in FOXP2 and other genes. We also discuss the finding that the processing of ancestral verb-noun compounds, which are typically used for (derogatory) naming and nicknaming, shows enhanced activation in the right fusiform gyrus area (BA 37), the area that is implicated in the processing of metaphoricity and imageability, but also in naming and face recognition, opening up an intriguing possibility that the enhanced face recognition in humans was facilitated by the early emergence of a simple syntactic strategy for naming.

**Discussion:**

The considerations in this paper are consistent with the hypothesis of a gradual gene-culture co-evolution of syntax and the brain, targeting cortico-striatal brain networks. It is also of note that a sound grounding in neurobiology of language should in turn inform syntactic theories themselves.

## Introduction

While it has certainly proven possible and fruitful to test how certain postulates of theoretical syntax are processed by the brain (see, e.g., Constable et al., 2004; Friederici et al., 2006; Grodzinsky and Friederici, 2006; Bemis and Pylkkänen, 2011; Friederici, 2011, 2017; Hagoort and Indefrey, 2014), it has also been suggested that this kind of approach has reached an impasse, ascribing it to the inherent mismatch in conceptual granularity between the concrete biological units of neuroscience, such as neurons, axons and dendrites, and the abstract postulates of linguistic theory, such as Determiner Phrase (DP) or Tense Phrase (TP; e.g., Poeppel and Embick, 2005; also Fedorenko and Kanwisher, 2009). With regards specifically to syntax, Flick and Pylkkänen (2020) have recently claimed that “although the construction of syntactic structures is considered a fundamental component of language processing, insights concerning its neurobiological basis have remained elusive. This may be due, in part, to the inherent difficulty of isolating incremental syntax from other components of language, such as semantic composition.”

Friederici (2011) summarizes the ways in which these problems have been addressed so far, with the common approaches addressing: (i) the contrast between sentence and word-list conditions, (ii) the effects of syntactic violations, and (iii) the effects of various manipulations of syntactic complexity (e.g., object vs. subject relative clauses). The results of this research have implicated several important left hemisphere regions, including the left anterior temporal lobe, the left posterior temporal lobe, the neighboring angular gyrus and temporo-parietal junction, as well as the left inferior frontal gyrus. The dominant view over the last several decades has been that Broca’s area (the posterior portion of the inferior frontal gyrus) plays a crucial role in the processing of syntax (see, e.g., Grodzinsky and Santi, 2008; Hagoort, 2014; Friederici, 2017; Zaccarella et al., 2017, among many others). Its role in the processing of specifically hierarchical syntax has also been reported in, e.g., Nelson et al. (2017), based on an experiment which found that brain activity increased with each successive word but decreased whenever several previous words could be compressed into a syntactic phrase, at which point an additional burst of activity was seen, primarily in the inferior frontal gyrus pars triangularis, and then the activation dropped. They interpret this finding to mean that the activity in the inferior frontal gyrus is responsible for the chunking of words into hierarchical structures.

On the other hand, Matchin and Hickok (2020) have questioned this dominant view. According to them, syntax processing depends on two main regions, the posterior portion of the inferior frontal gyrus, but also the posterior middle temporal gyrus, where Broca’s area is mostly involved in production, rather than comprehension, with some involvement in the comprehension of complex syntax only. That said, according to Friederici (2011), “while valuable, the contribution of these findings to a neurobiological account of syntax is limited by how well the manipulations isolate syntax, *per se*.” The approach we outline here is aimed to address these challenges, by suggesting a novel neurobiological way of dealing with the incremental nature of syntax, as well as of isolating it from semantic composition.

As is the case with the references mentioned above, here we focus on the syntax of human language(s), whose complexity can be attributed partly to the multiple layers of structure, creating a hierarchy of functional projections, as illustrated and elaborated in section 3. But human grammars rely not only on the combinatorial processes that combine words into phrases and sentences, but also on a host of grammatical words or morphemes (e.g., *of, will, −ed, −ing, and, or, for, to, if, when*), which serve a variety of abstract functions, including building and holding together these multiple layers of structure. As will be illustrated in detail in the sections to follow, not all phrases and sentences in present-day languages are created equal when it comes to the number and nature of the layers of structure and grammatical words, and this variability in complexity offers a fertile ground for evolutionary considerations regarding the emergence of syntax, seen both as a cultural phenomenon and as a genetic adaptation.

## A new perspective for neurolinguistic research on syntax

Our first main argument is that neurolinguistic research on syntax needs to be informed not only by our current knowledge of syntax as manifested in the world languages, but also by the considerations of how syntax changes over time and, in particular, how it might have evolved in the species (e.g., Progovac, 2015; Progovac, 2010b; Progovac, 2010a), or, more precisely, how it co-evolved in a gene-culture feedback loop with the brain. In this respect, we argue for decomposing syntactic phenomena also in terms of more ancestral vs. more modern components, and for devoting more attention to the former, in order to complement current neurolinguistic research. Modern forms of human language syntax can indeed be expected to result from the interplay between many assorted representations and computations, and ultimately, from complex interactions between many brain regions: hence the intricate neurolinguistic picture, as sketched in the previous section. Incidentally, this intricacy and the evolutionary novelty of modern syntax seem to account for its increased susceptibility to ontogenetic and acquired perturbations, as recently evolved neuronal devices usually exhibit a reduced resilience and weaker damage protection mechanisms (see Toro et al., 2010; or Pattabiraman et al., 2020, for general discussions). By contrast, simpler, ancestral forms of syntax are expected to depend on more specific, less distributed neuronal devices. Accordingly, focusing on them should enable us: (i) to find (and facilitate the study of) the components of syntax that are more tractable by neuroscience; (ii) to find more continuity with other species, this including better animal models for studying syntax in the brain (as older attributes are expected to be shared with, and more tractable in animals);^1^ and (iii) to improve our understanding of the etiology of conditions entailing problems with syntax (because, as noted, evolutionarily newer components are expected to be more susceptible to damage). Ultimately, this approach should provide more robust linking theories between the neuroscientific method and theoretical postulates about syntax, which can also have ramifications for linguistic theory: ideally, one’s theories about language should be compatible with how the brain processes language in real time.

In this respect, our focus here is on the theoretical postulates of Minimalism (e.g., Chomsky, 1995; Adger, 2003; Citko, 2011), the syntactic framework that is widely adopted, especially in the United States, and which has been used extensively in neuroimaging studies, including the studies mentioned in the Introduction. Also, our previous work on the reconstruction of early grammars, as well as the neuroimaging experiments designed to test the predictions of that approach, have relied on this framework, which provided enough specific detail to allow such testing. The reconstruction based on this framework also reveals a common denominator regarding cross-linguistic variation, thus engaging yet another important dimension for language evolution. Having said that, we believe that each linguistic framework sees only part of the complex picture, and that testing the predictions of other frameworks will be necessary to complete the picture, as different approaches illuminate different aspects of language. To take one example, Jackendoff and Wittenberg (2017) have proposed a stage in language evolution that would have involved what they call linear grammar, that is, simple stringing of words, where the sound and meaning were mapped directly, without any tools of (modern) syntax (see also Wittenberg and Jackendoff, 2023). As they point out, modern languages (both their typical and atypical manifestations, the latter found in various clinical conditions) exhibit instances of linear ordering playing a direct role in interpretation, and these include agent before patient ordering, and cause before effect ordering. We see this approach as complementary to the approach we explore here: while Jackendoff and Wittenberg’s approach considers possible stages in language evolution without syntax, our approach focuses specifically on how syntax evolved, in a binary, layered, step-by-step fashion. As pointed out in Progovac (2019), these different approaches to language evolution can prove to be highly synergistic (including also Heine and Kuteva’s, 2007 work on grammaticalization, mentioned below).

The second important argument we wish to advance, related to the first, is that neurolinguistic research on syntax needs to be more informed as well by other uses of syntactic structures beyond the ones conveying complex propositional content, which have been the main concern for researchers for decades. To a great extent, language is used for expressing emotions, socializing, or persuading others Moreover, it can be expected that these uses are evolutionarily older and predate the transmission of propositional content (see also footnote 7), as has been argued by others when it comes to the continuity with the other species (e.g., Hilliard and White, 2009; Podlipniak, 2022; see also Benítez-Burraco et al., 2021 for a possible trajectory of persuasive abilities during human evolution, from crude to sophisticated). In contrast, propositional uses are expected to be much more reliant on complex, more recently evolved forms of syntax. Consequently, we wish to argue that neurolinguistic research on syntax should be supplemented and enriched by the consideration of utterances, chunks, formulae, and the like used for fulfilling non-propositional functions of language.

Taking these “marginal” syntactic phenomena into account becomes even more relevant if our syntactic abilities co-evolved with our brain, in a gene-culture feedback loop with each other, as well as possibly with some other nonlinguistic phenomena, specifically, the management of emotions/aggression, as we will elaborate below. We use the term co-evolution here in the sense of gene-culture interaction, where cultural innovations bring about, and later complexify, grammars, which in turn exerts pressure on the brain by way of natural selection, favoring those individuals whose brains are better equipped to process and make use of such innovations, including syntactic hierarchy.^2^ Ultimately, this feedback loop yielded a species whose syntactic abilities are automatic and instinctive, and who is genetically predisposed to acquire language. Advocating co-evolution of language and emotions, Jablonka et al. (2012: 2152) have also ascribed an active, causal role to language in human evolution, stating that the emergence of some genuinely new cultural/linguistic practices “drove a process of genetic accommodation of both general and language-specific aspects of cognition” (see also Deacon, 2003; Clarke and Heyes, 2017).^3^

In the rest of the paper, constantly keeping in mind our two main arguments, we discuss different types of evidence supporting the approach we advocate for, as well as outline some specific future prospects and hypotheses to be tested in this regard, also taking into account cognitive disorders.

## More hierarchical vs. less hierarchical, ancestral syntactic constructions

As noted in the previous sections, studies on syntax have converged on the conclusion that it depends on a large circuit that involves different cortical areas going beyond Broca’s area, but also subcortical structures, including the striatum in the basal ganglia (e.g., Gibson, 1996; Lieberman, 2000, 2009; Teichmann et al., 2005; Vargha-Khadem et al., 2005; Ullman, 2006; Ardila et al., 2016a, 2016b; Progovac et al., 2018; see Murphy et al., 2022 for a recent review). Consider, however, that with few exceptions (see below), this detailed picture of syntax in the brain has resulted from studies using as stimuli complex, hierarchical syntactic objects, such as transitive or ditransitive sentences, embedded clauses, passives, etc. Consider also that whereas some components of this complex circuit are evolutionary old, some others can be regarded as more recent developments. For instance, there is evidence that neuronal connectivity of the Broca–basal ganglia network has been significantly enhanced relatively recently in evolution, in the line of descent of humans (and Neanderthals), implicating recent mutations in *FOXP2* and other genes (see, e.g., Enard et al., 2009; Hillert, 2014; Dediu, 2015). In other words, some recent mutations in these genes have enhanced the density of neuronal connectivity in this brain network. This finding is consistent with the hypothesis that this network co-evolved with the gradual (cultural) emergence and then complexification of language/syntax, in a culture-gene feedback loop. What is more, as will become relevant later in the discussion, this network overlaps significantly with the networks responsible for the suppression of reactive aggression. Overall, under this approach, we can expect that more hierarchical syntax is processed differently by the brain, when contrasted with the processing of less hierarchical, ancestral syntactic constructions (see Progovac, 2010a, 2015). Ultimately, this approach is able to address the difficulty of testing incremental syntax, mentioned above (Flick and Pylkkänen, 2020).

Against this background, Progovac et al. (2018) performed fMRI experiments testing how flatter syntactic structures (e.g., small clauses of the kind: *Problem solved; Crisis averted; Obama elected; Dishes done; Body found; Signature needed*) are processed by the brain, in contrast to their more hierarchical counterparts (i.e., full sentences of the kind: *The problem was solved; The crisis was averted; Obama was elected; The dishes are done; A body was found; A signature is needed*), the latter, but not the former, featuring a full expression of the abstract syntactic postulates referred to as TP (Tense Phrase) and DP (Determiner Phrase) (e.g., Chomsky, 1995; Adger, 2003; Citko, 2011).^4^ This study also compared flat(ter) verb-noun compounds (e.g., *kill-joy*, *cry-baby*), with their more hierarchical counterparts (e.g., *joy-kill-er*, *boot-lick-er*).^5^ The main finding of Progovac et al.’s (2018) study was that less hierarchical small clauses resulted in reduced activation in the left Broca’s area and right basal ganglia, whereas flat(ter) compounds resulted in increased activation in the inferior temporal gyrus and the fusiform gyrus (Brodmann’s areas 37/19), regions not typically thought to be relevant for the processing of syntax. Importantly for our concerns here, both of these constructions (small clauses and compounds) feature the foundational ingredients of modern sentences, i.e., verbs and nouns, which are at the heart of all human languages. When it comes to the processing of small clauses vs. full sentences, the finding is consistent with the hypothesis that syntactically simpler, more ancestral structures (small clauses) could have been processed with brains exhibiting less dense neuronal connectivity, specifically in the Broca’s-basal ganglia network. The relevance of the fusiform gyrus in human evolution will also be discussed below.

Overall, such flat(ter) structures have been proposed to have predated their hierarchical counterparts in language evolution (Progovac, 2015; see also Jackendoff, 1999). Based on some crucial aspects of syntactic theory, but also on the patterns of cross-linguistic variation in syntax as found in modern languages, Progovac (2015, 2016, 2019) has advanced a detailed reconstruction of changes in sentential structure during language evolution. Very roughly speaking, in the Minimalist framework for syntax, a full sentence in, e.g., English consists of these basic syntactic layers, and possibly additional ones, too (e.g., Adger, 2003; Citko, 2011):

(1) TP > vP > SC/VP.
  [Here, TP refers to the Tense Phrase; vP to a second layer of the verb Phrase, which accommodates transitivity; VP refers to the basic Verb Phrase; and SC to a Small Clause].

These 3 basic layers can accommodate a full transitive sentence such as ‘Maria will grow corn,’ where the structure in this framework is built gradually, in a binary fashion, starting from the bottom layer (as if the building of the modern sentence retraces its evolutionary steps):

(2) Maria will grow corn.
  - [_VP_ grow corn] →
  - [_vP_ Maria [_VP_ grow corn]] →
  - [_TP_ Maria will [_vP_ Maria [_VP_ grow corn]]].
  [Here, the subject ‘Maria’ moves from the vP layer, where it is initially merged, to the TP layer, as dictated by the principles of English syntax; see, e.g., Adger, 2003.]

With this in mind, Progovac (2015, 2016) has reconstructed the bottom layer of this syntactic hierarchy, basically the intransitive verb phrase (or small clause) layer, involving a verb and just one noun (phrase), as the proxy for the initial stages of ancestral grammars.^6^ In this respect, the hierarchy of sentential structure in (1) provides a straightforward method of reconstructing the earliest, foundational stage of grammar, as well as the necessary precision and detail which comes with every additional layer. In other words, by removing the higher layers of structure, we can see exactly what would be missing in the earliest layer.

In addition to still “living” inside the full sentences, this kind of ancestral grammar is approximated (in certain relevant respects) by modern root small clauses and verb-noun compounds across different languages, as illustrated above for English (see also below). It is important to point out that these proxies of earlier grammars are just that: proxies or approximations, as they certainly cannot be seen as identical to the language produced at the earliest stages (for an extensive discussion of how these “fossil” structures need to be seen, and how they can be used in current research, see Progovac, 2019). For example, verb-noun compounds in some languages, including Serbian, exhibit some morphology, in particular some (ancient) imperative-like morphology on the verb, which shows that these combinations truly involve verbs, participating in constructing mini sentences (or small clauses).^7^ Similarly, English small clauses mentioned above (such as *Problem solved*, *Dishes done*) involve passive participles of the verbs, the morphology which certainly is not expected to have existed when language just started to emerge. Still, such root small clauses can be seen as approximations of these earliest grammars in that they lack at least two modern layers of structure, i.e., TP and DP (i.e., the auxiliary verb/tense and the determiner), which are required in complete sentences in English (*cf. The problem is solved*. *The dishes are done.*)

The above line of research (particularly, Progovac and Locke, 2009 and Progovac, 2015) also finds that among the preserved verb-noun compounds across languages derogatory ones predominate when they refer to humans (*kill-joy, cry-baby*; more examples and details to follow). In fact, it seems that this compound type is especially suitable for naming purposes more generally (including naming animals and plants: *rattle-snake*; *tumble-weed*), the importance of which is discussed below. This suggests some permanence in these labels, which would have given them even more importance in the deep evolutionary past, especially if they were used to name individuals, effectively amounting to nicknames. This compounding strategy is no longer productive in English or Serbian, as these languages have developed a more layered compound type (e.g., *joy-kill-er* in contrast to *kill-joy*), but the compounds that are preserved in this form tend to refer to people in a derogatory fashion, implicating verbal aggression (see the discussion in the following section). In this respect, this type of simpler, evolutionarily older grammatical structures can be linked to more emotional, non-propositional uses of language, in comparison to more complex, evolutionarily newer components, which seem to be better specialized for propositional uses of language. In our view, relying on this kind of specific linguistic data and detail, including usage-based approximations, is crucial for advancing testable hypotheses about the gene-culture (co-)evolution of human language and the brain.

As introduced above, this ancestral proto-grammatical strategy, at least what is preserved of it in modern languages, does seem to be particularly fit for *naming*. According to Weekley (1916) book titled *Surnames* devoted specifically to these compounds, there were thousands of such verb-noun compounds created in medieval times, constituting an “expressive way of *naming*,” but such compounds tend not to get preserved in dictionaries or grammar books because they often show “unquotable coarseness” (Weekley, 1916). Regarding French, for Darmesteter (1934: 443), the artistic beauty and richness of these compounds is inexhaustible: “it would be well could French poets again make use in lofty poetry of this class of *epithets*, for they may attain Homeric breadth…” Mihajlović (1992), who devoted his career to collecting over 500 Serbian people and place *names* in the form of these compounds, calls them condensed compositions which pack in them … frozen fairy tales, proverbs, and ancient wisdoms and metaphors (1992: 8–9). In other words, the examples preserved across languages are primarily used for naming, and they tend to be highly expressive and metaphorical, as well as (playfully) derogatory when referring to humans. Such compositions make use of meager means (basic, crude vocabulary and rudimentary syntax) to express highly abstract, novel concepts, exactly what would have been needed and appreciated at the earliest stages of language, ultimately contributing to creating a species that values cognitive contest over physical fighting.

The following examples from English (3) and Serbian (4) illustrate this strategy (see Progovac, 2015, 2016 for many more colorful examples from these and other languages):

(3) kill-joy, turn-skin, turn-coat, hunch-back, wag-tail, tattle-tale, scatter-brain, cut-throat, mar-wood (bad carpenter), heck-wood, busy-body, cry-baby, break-back, catch-fly (plant), cut-finger (plant), fill-belly (glutton), lick-spit, pinch-back (miser), shuffle-wing (bird), skin-flint (miser), spit-fire, swish-tail (bird), tangle-foot (whiskey), tumble-dung (insect), crake-bone (crack-bone), shave-tail (shove-tail), wipe-tail, wrynge-tail, fuck-ass, fuck-head, shit-ass, shit-head.
(4) ispi-čutura (drink.up-flask—drunkard); guli-koža (peel-skin—who rips you off); cepi-dlaka (split-hair—who splits hairs); muti-voda (muddy-water—trouble-maker); vrti-guz (spin-butt—fidget); pali-drvce (ignite-stick, matches); jedi-vek [eat-life = one who constantly annoys]; kosi-noga [skew-leg = person who limps]; mami-para [lure-money = what lures you to spend money]; podvi-rep [fold-tail = someone who is crestfallen]; priši-petlja [sow-loop = who clings onto another]; probi-svet [break-world = wanderer]; raspi-kuća [waste-house = who spends away property]; kaži-prst (say-finger = index finger); jebi-vetar (fuck-wind—charlatan); deri-muda ‘rip-balls’ (place name, a steep hill); gladi-kur ‘stroke-dick’ (womanizer); kapi-kur ‘drip-dick’ (name of a slow water spring); plači-guz ‘cry-butt (crybaby).

As pointed out in, e.g., Darwin (1872), strong emotions expressed in animals are those of lust and hostility, and they may have been the first verbal threats and intimidations uttered by humans. If there is some truth to that, then creative proto-syntactic two-slot compositions (well-suited for playful insult) would have provided a rather graceful transition from other primate cognitive abilities and preoccupations to human language.^8^ But, crucially, when these two-slot combinations started to be used, they could have been used for so many other functions as well, including to issue commands (*Eat apple, Run Kanzi*) or to make observations (e.g., *Fall apple, Cry baby*). While the earliest, most adaptive functions may have been non-propositional and manipulative, the transition to propositional language did not have to be abrupt. In fact, as the above examples show, the line between issuing a command (*Cry baby!*), uttering an insult (*Cry-baby!*), and making an observation (*Cry baby*) is not a sharp one at all.

Although, as noted, this has not been a common approach to the processing of syntax in the brain, other researchers have studied the processing of simpler (two-word) syntactic constructs, most notably Pylkkänen (2019) and Bemis and Pylkkänen (2011), including adjective-noun combinations and determiner-noun combinations. The main finding of this research was that structures with determiners involve more (hierarchical) syntactic processing than structures with adjectives, which already suggests that not all two-word combinations are created equal. In this respect, Progovac et al.’s (2018) study mentioned above is complementary, as it also considers two-word constructions, but instead of considering adjectives or determiners, this study considers verbs and nouns, which constitute the crucial ingredients of modern sentences.^9^ This research thus goes a step further, focusing on combinations of the two-word type that have been argued to be precursors to modern sentences in language evolution (see above). In addition, Progovac et al. (2018) study considers minimal pairs using the same content words (e.g., *Signature needed*, vs. *A signature is needed*), involving pairs of free-standing utterances which mean basically the same thing in the context of the experiment. As such, this study is well-positioned to control for semantic factors, which, as noted in the Introduction, is notoriously difficult, but necessary when aiming to identify the neurobiological substrate of syntax vis-à-vis semantic composition. While it may never be possible to completely isolate syntax from semantics in neuroimaging studies, Progovac et al. (2018) study comes closer to that goal than, e.g., studies comparing combinations with adjectives vs. determiners, as the latter studies necessarily involve completely different basic words, each with their own semantic baggage (consider, e.g., the contrast: *the cats* vs. *small cats*).^10^ It certainly seems easier to control for semantic factors in pairs such as *Dishes done* vs. *The dishes are done*, than in pairs such as *the cats* vs. *small cats*.

The finding that less hierarchical syntax without TPs and DPs demands less activation in the Broca-basal ganglia network can be partly explained by the fact that the neuronal connectivity of this network has been significantly enhanced only in the recent evolution, as mentioned above. Given that TPs and DPs are rather recent innovations in the gradualist approach to syntax considered here, their processing is expected to require more connectivity in this brain network.^11^ While it may not be possible to directly isolate the abstract notions such as TPs or DPs in the brain (the apparent granularity mismatch), it is still possible to compare the processing of more modern constructions which feature these categories, i.e., tense and determiners (*A signature is needed*) with corresponding small clauses which lack them (*Signature needed*), and the findings in Progovac et al. (2018) provide proof of concept that such studies can yield significant results. This is thus a way to circumvent the thorny granularity mismatch problem, as introduced in the Introduction, i.e., the apparent mismatch between the abstract categories of theoretical syntax and the concrete biological units of neuroscience. It is in this sense that studying approximations of ancestral syntax may help us achieve a better characterization of how the brain processes syntactic structures in general. For these reasons, in our view, further and more extensive studies of this kind can enrich and improve the current understanding of syntactic processing by the brain, as well as shed light on the language-brain gene-culture co-evolutionary spiral.

## The fusiform gyrus: faces and names

Next, consider Progovac et al. (2018) finding regarding the processing of flat(ter) verb-noun compounds, implicating the inferior temporal gyrus and the fusiform gyrus, specifically right Brodmann’s area (BA) 37 (a region located more exactly in the posterior portions of the fusiform gyrus and the inferior temporal gyrus of the temporal lobe). In addition to their more familiar functions mentioned below, these areas, in particular BA 37, are also implicated in face perception and recognition (Kanwisher et al., 1997; Grill-Spector et al., 2017; Rapcsak, 2019; Barton, 2022). In fact, as pointed out in, e.g., Weibert and Andrews (2015), face recognition seems to be facilitated primarily by the right (as opposed to left) BA 37, which was also the case with the processing of verb-noun compounds in the experiment mentioned above.

It is of note that the fusiform gyrus area has been implicated in semantic language processing, including tasks involving *naming*, concreteness, and metaphoricity (see a recent meta-analysis by Ardila et al., 2015). This is consistent with the possibility that regions that help process these more elementary components of syntax in fact perform some basic computations not restricted to language, in line with Poeppel and Embick’s (2005) claim that “differently structured cortical areas are specialized for performing different types of computations, and […] some of these computations are necessary for language but also for other cognitive functions.” Accordingly, further studies along the lines of those in Progovac et al. (2018) may enable us to identify more evolutionary continuity for syntax, as other primates are sensitive to faces, too. At the same time, because primates show reduced abilities for individual face recognition in comparison to humans, as they process faces more holistically, some subtle neuroanatomical changes might have happened in our species to bring about such differential processing abilities (see Rossion and Taubert, 2019), including potentially those that are relevant for syntax. In fact, the involvement of the fusiform gyrus area in humans in both face recognition and in naming may not be a coincidence, opening up an intriguing possibility that the enhanced face recognition in humans was facilitated by the early emergence of language, specifically the early proto-grammatical means of naming people (as introduced in the previous section), which is essentially an act of associating linguistic labels with people’s faces. This is certainly a hypothesis worth exploring further.

Additionally, the fusiform gyrus region seems to be a primary locus of noun-verb classification (Boylan et al., 2014). Interestingly, according to Boylan et al. (2014), word-form estimates for differentiating between nouns and verbs in the fusiform gyrus areas may arise not from semantic aspects (e.g., imageability), but from syntactic cues, because this area seems unable to classify nouns vs. verbs when they are presented isolated.^12^ Related to that, the fusiform gyrus region has been associated as well with the ability to remember sentence fragments (i.e., basic syntactic objects), compared to word lists, and can thus be argued to contribute to the generation of (syntactic) memory chunks (Bonhage et al., 2017). As acknowledged by Makuuchi and Friederici (2013), the fusiform gyrus area is at the bottom of a hierarchy of computational resources recruited for processing increasingly complex syntactic structures.

In summary, the region highlighted in research addressing the processing of structurally simpler, ancestral syntactic objects (e.g., Progovac et al., 2018), also happens to be a brain area involved in naming and face recognition, as well as in semantic processing and visual processing more generally, with structural and functional homologs in other species. Accordingly, studying the role of this and other similar regions has a potential to provide a smoother entrance to the primitives of the syntax-semantics interface, as well as to find evolutionary continuity with the cognitive abilities exhibited by other species, which is a topic under much debate (see Beckers et al., 2017; Engesser and Townsend, 2019; Suzuki et al., 2020; Schlenker et al., 2023 for recent discussions).

## More than propositional content

With regards to our second main argument, namely the need to consider other, non-propositional uses of syntax and, eventually, synergistic evolution of syntax and other nonlinguistic phenomena, we find it of particular interest that the cortico-subcortical networks that are implicated in the processing of hierarchical syntax, as characterized above, are functionally connected to, and partially overlap with, the networks implicated in the suppression of aggression in humans (and other species). To take one example, Lischinsky and Lin (2020) have found, specifically, that the suppression of aggression demands an increased control of the striatum, among other subcortical regions, by the prefrontal cortex. This conclusion is supported by the finding that diseases resulting from striatal dysfunction, such as Parkinson’s and Huntington’s diseases, not only feature problems with hierarchical syntax (Rosenblatt and Leroi, 2000; Moro et al., 2001; Teichmann et al., 2005; Teichmann et al., 2008; Newman et al., 2010), but also feature increased reactive (i.e., automatic) aggression, the latter typically accompanied by higher levels of stress and anxiety (Savage, 1997; Rosenblatt and Leroi, 2000).^13^

As with the striatal diseases mentioned above, the same comorbidity between atypical syntax and elevated aggression levels can be observed in conditions like autism spectrum disorder (ASD) and Tourette’s Syndrome (TS). People with ASD tend to exhibit atypical uses of complex syntax (e.g., accusative clitics, Prévost et al., 2018; relative clauses, Durrleman et al., 2015; *wh*-questions, Prévost et al., 2017; passives, Durrleman et al., 2017; Ambridge et al., 2021; or embedded clauses, Silleresi et al., 2018), as well as elevated reactive aggression (Langen et al., 2009; Hill et al., 2014). Likewise, TS has been reported to exhibit elevated reactive aggression (Ganos et al., 2014), as well as uncontrollable verbal aggression (coprolalia) in some TS individuals. In addition, both people with ASD (Baron-Cohen et al., 2009; Ward et al., 2017) and with TS (Walenski et al., 2007) have been reported to exhibit hyper-systemizing, i.e., a rigid application of rules even when they should not be applied, this being suggestive of an increased striatal function (see Benítez-Burraco and Progovac, 2023 for a unified treatment of linguistic and behavioral symptoms of rigidity in autism). Importantly for our argument here, both conditions feature interneuron dysfunctions that give rise to an altered inhibition of specific cortico-striatal circuits, resulting, specifically, in reduced control of striatal activity by cortical structures (Rapanelli et al., 2017; see also McBride and Parker, 2015; Nelson and Valakh, 2015; Traynor and Hall, 2015). In spite of these findings, and although emotive and expressive uses of language, including verbal aggression, are extremely common, neurolinguistic research hardly ever addresses them (Shanahan, 2008). Likewise, the role of the brain mechanisms controlling aggression, and emotions more generally, has played only a minor role in discussions about the evolution of language.

In this respect, it is of special interest that the postulated approximations of early stages of language evolution, specifically verb-noun compounds, feature both emotional, derogatory language (i.e., verbal aggression) and simpler, flat(ter) syntax, as demonstrated above with the examples (3–4). Evolutionarily, this new derogatory language would have provided especially useful means of verbal/cognitive contest, proving to be highly adaptive, as it would have favored the gradual replacement of reactive physical aggression with verbal aggression, and with verbal behavior more generally, which is less harmful than physical fighting.^14^ This evolutionary development has been proposed to result from the feedback loop between our increased Human Self-Domestication (HSD) and the cultural emergence of the new forms of syntax/language, at first flat and rudimentary, and later increasingly more sophisticated and hierarchical (see Progovac and Benítez-Burraco, 2019; Benítez-Burraco and Progovac, 2021 for details). A key ingredient of the HSD hypothesis is a reduction in reactive aggression, a hallmark of the human behavioral phenotype (Hare et al., 2012; Rilling et al., 2012; Hare, 2017).^15^ If our proposal is on the right track, then our brains evolved, in part, to accommodate the processing of increasingly complex syntax (and language more generally), which, in the process, yielded a less reactive phenotype (specifically a phenotype favoring cognitive contest over physical fighting), and perhaps also a phenotype better able to distinguish human faces.

## Conclusion and future prospects

In sum, our main argument in the paper has been that neurolinguistic research on syntax would benefit greatly by expanding its scope to include evolutionary considerations. This entails considering flat(ter) syntactic structures, in particular those which can be hypothesized to approximate more ancestral forms of syntax, as well as non-propositional, emotional uses of language/syntax, which can be considered to be older functions of language. Overall, the primary goal of our proposal is rendering the postulates of syntax more tractable by neuroscience, by decomposing syntax into its evolutionary primitives, and then tracking how these primitives are processed in contrast to incrementally more complex constructions. Given how well-suited human brains are for fast and effortless processing of syntax, it stands to reason that our brains adapted and adjusted to the (gradual) emergence of syntax in the course of human evolution, and that these adjustments should be detectable by neurolinguistic methods. We also wish to highlight that neurobiological considerations of syntax are not only ways to test evolutionary hypotheses, but that sound grounding in neurobiology of language should also ultimately inform linguistic theories themselves, as well as contribute to a better understanding of the enhancements in various other cognitive abilities in human evolution.

With regards to specific ways of testing and further refining our hypothesis, one fruitful way would be to examine the processing of proto-syntactic structures vs. more modern hierarchical structures not only in typical populations, but also in individuals with ASD or TS, with the goal to determine if they show a different type of activation in the Broca-basal ganglia network, as well as other relevant brain regions, such as the fusiform gyrus area. If our hypothesis is on the right track, we can expect a more focused reliance on striatal networks in these two neurodiverse groups. In addition, one can test the processing of non-propositional, emotional uses of language, including positive and negative emotions, in both neurotypical populations and in populations with ASD or TS. For ASD and TS, we would predict atypical functional overlaps between the networks involved in the processing of syntax and the circuits involved in controlling aggression. Our proposal also opens up a possibility to test the involvement of the fusiform gyrus region in naming/nicknaming and face recognition, specifically trying to determine to what extent these two processes interact/overlap in neurotypical and atypical populations. We consider this a special strength of our approach: the ability to advance specific testable hypotheses, with the reported experimental findings not only providing proof of concept that this can be done, but also identifying new frontiers for testing.

## Data Availability

The original contributions presented in the study are included in the article/supplementary material, further inquiries can be directed to the corresponding author.

## Footnotes

^1^Finding more evolutionary continuity with the combinatorial abilities of other species should result in more parsimonious accounts of the emergence of human syntax, in contrast with narratives that consider it as a sort of evolutionary punctuation (e.g., Berwick and Chomsky, 2016).

^2^One familiar example of gene-culture co-evolution involves lactose tolerance, where the cultural invention of farming and consuming milk resulted in the genetic accommodation through natural selection, favoring those individuals who were better able to benefit from this innovation, and ultimately spreading to the entire populations in, e.g., some parts of Europe (e.g., Stone and Lurquin, 2007).

^3^Originally, Darwin (1872: 634) attributed an active, causing role to language in important changes in the brain and our cognitive abilities, stating that “a great stride in the development of intellect will have followed, as soon as … language came into use … The largeness of the brain in man … may be attributed in chief part to the early use of some simple form of language” (see also Pinker and Bloom, 1990; Deacon, 2003). Of note is that Darwin envisioned a gradual evolution of language, where already the simple forms of language exerted influence on the brain, which is consistent with the feedback loop approach that we advocate here, as well as in our previous work.

^4^The small clauses of the type illustrated in the text certainly involve syntax, combinations of verbs and nouns; while some of them can be semi-formulaic, this combinatorial strategy is clearly productive, as it is found in novel compositions, such as *Obama elected*. The claim here is that the earliest forms of syntax were more like *Body found* than *A body was found*. The question here is exactly the following: what kind of brain mechanisms are/were needed to process these simpler, more compact compositions, and how did human brains evolve to be able to accommodate the compositions involving abstract functional categories and layers of grammar.

^5^As routinely acknowledged by linguists, there is no sharp dividing line between morphology (the structure of words) and syntax (the structure of phrases and sentences). The famous insight by linguist Tom Givón that “Today’s morphology is yesterday’s syntax” is certainly applicable to these verb-noun compounds. The distinctions possible to make in English (among *kill-joy, joy-killer, the killing of joy, They are killing my joy*) are only due to a variety of (recently evolved) morpho-syntactic devices, but they all have a verb and a noun layer as their foundation. The claim here is that the earliest stages only afforded a simple combination consisting of just one verb and one noun, akin to the compound *kill-joy*, which could be interpreted in a variety of ways.

^6^Languages of the world differ significantly in how they express, e.g., transitivity. The small clause foundational layer provides an excellent common denominator, i.e., common platform, from which to evolve transitivity across languages in diverging directions, including nominative-accusative patterns, ergative-absolutive patterns, and serial-verbs patterns (see Progovac, 2015, 2016). This reconstruction thus meaningfully engages another important dimension of language evolution, that is, variation across languages.

^7^As discussed in detail in Progovac (2010b, 2015), verb-noun compounds in Serbian feature an ending on the verb (*i/j*) that coincides with the ending used with what is synchronically the imperative form, as in, e.g., *jed**i**-vek* [eat-life, somebody who annoys] (e.g., Stevanović, 1956). This kind of imperative is also found in some other frozen expressions in Serbian, such as optatives (the grammatical mood for expressing wishes), such as *Pomoz**i** Bog* (Help God!; in English: *God Forbid*! *Long Live the King*). It is relevant in this respect that the Slavic imperative mood descended from the optative mood expressing wishes, often indistinguishable from commands, which in turn descended from the ancient Proto-Indo-European (PIE) injunctive mood (e.g., Kiparsky, 1968; Kerns and Schwartz, 1972: 23; Stevanović, 1974). Gonda (1956: 36–7) points out that any attempt exactly to translate the injunctive categories into a modern Western idiom is doomed to fail, given “the vagueness in meaning and the great, and in the eyes of modern man astonishing, variety of its functions.” According to Kuryłowicz (1964: 21), the injunctive, a tenseless verbal form, was the only mood in earliest PIE, and it was initially an unmarked mood, encompassing both non-indicative and indicative functions. After the indicative mood emerged as a separate grammatical category, specializing for expressing propositions, the injunctive mood was left to specialize for non-indicative, “irrealis” functions, expressing wishes, commands, exclamations, etc. It is also important for our considerations here that even in historical times one can see that indicative mood may not have been a distinct mood early on, and certainly not the dominant mood, suggesting that expressing propositions unambiguously was not the high priority in ancestral language.

^8^It is worth noting that other primates seem capable of simple two-slot combinations (with no subject/object differentiation), such as *hide peanut* and *hide Kanzi* (see, e.g., Greenfield and Savage-Rumbaugh (1990: 161) regarding bonobo Kanzi). Moreover, according to Patterson and Gordon (1993), gorilla Koko is not only capable of producing novel two-word metaphorical combinations, but also of insult and playfulness. Our approach thus reveals some continuity with other species.

^9^In this respect, many languages do not have determiners such as articles (*a, the* in English), and many have been argued not to have a separate category of adjectives. For this reason, the studies that involve adjectives and determiners, while certainly valuable in other respects, cannot shed direct light on the early evolution of syntax, whereas the studies that involve nouns and verbs have a much better chance at that, as these categories are shared across languages, and provide the most important building blocks for sentences across modern languages. The reader is also referred to Heine and Kuteva’s (2007) reconstruction of word categories based on grammaticalization processes, which establishes nouns and verbs as the earliest word categories in language evolution, and the emergence of articles as a much later development.

^10^Full counterparts of root small clauses feature a Tense Phrase which allows expression of past and future tenses (e.g., *The problem will be solved ten years from now*), the expansion not available to the small clause counterparts (??*Problem solved ten years from now*). This reinforces the conclusion that there are no null categories of Tense in the small clause counterparts (see Progovac, 2015 and references cited there). Even though *The problem is solved* can in principle mean different things than *Problem solved*, in the context of the experiment, their default interpretations were the same, or at least as close in meaning as possible to achieve in an experiment of this kind.

^11^By recent we mean in the thousands or tens of thousands of years, and not in the hundred thousand years. The dating of the emergence of language is still quite controversial (e.g., Dediu and Levinson, 2013), but see Progovac and Benítez-Burraco (2019) for an attempt that takes into account the considerations outlined in this paper, as well as the fossil and archaeological records.

^12^This can be seen as supporting Lenneberg’s (1967) view that differentiation among syntactic categories such as nouns vs. verbs is determined on the basis of their functioning in syntactic configurations. For a detailed recent discussion about how the emergence of grammatical categories is intertwined with the emergence of syntax, the reader is referred to Benítez-Burraco et al. (2023).

^13^It is certainly true that many other functions also rely on the striatal networks. Our argument is not that these networks are solely there to suppress aggression, or to enhance syntax, but that the enhancement in neuronal connectivity in striatal networks was essential to both of these processes, which is consistent with the hypothesis of their synergistic evolution, or co-evolution, in the sense of two distinct traits evolving together by reciprocally influencing each other (see, e.g., Jablonka et al. for the co-evolution of language and emotions).

^14^Our proposal differentiates reactive aggression from premeditated, proactive aggression, the latter actually gradually increasing in humans, as discussed in, e.g., Wrangham (2018), Progovac and Benítez-Burraco (2019), and Benítez-Burraco and Progovac (2020). It is relevant in this respect that the derogatory uses of language, found especially among the verb-noun compounds approximating ancestral stages of syntax, are well-suited to replace reactive aggression, i.e., the more ancient forms of aggression, rather than premeditated aggression.

^15^Other typical “domestic” features found in modern humans arguably include physical (smaller skull/brain, neotenic features, loss of hair, etc.) and behavioral features (high(er) degree of socialization, friendliness, teaching, etc.)
